# The Use of Antioxidant Potential of Chokeberry Juice in Creating Pro-Healthy Dried Apples by Hybrid (Convection-Microwave-Vacuum) Method

**DOI:** 10.3390/molecules25235680

**Published:** 2020-12-02

**Authors:** Jolanta Kowalska, Agata Marzec, Ewa Domian, Sabina Galus, Agnieszka Ciurzyńska, Andrzej Lenart, Hanna Kowalska

**Affiliations:** 1 Department of Technology and Food Evaluation, Warsaw University of Life Sciences, Institute of Food Sciences, 159c Nowoursynowska St., 02-776 Warsaw, Poland; 2 Department of Food Engineering and Process Management, Warsaw University of Life Sciences, Institute of Food Sciences, 159c Nowoursynowska St., 02-776 Warsaw, Poland; agata_marzec@sggw.edu.pl (A.M.); ewa_domian@sggw.edu.pl (E.D.); sabina_galus@sggw.edu.pl (S.G.); agnieszka_ciurzynska@sggw.edu.pl (A.C.); andrzej_lenart@sggw.edu.pl (A.L.); hanna_kowalska@sggw.edu.pl (H.K.)

**Keywords:** apple, apple chips, freeze-drying, puffing, biocomponents, antioxidant activity, vitamin C

## Abstract

The visible trend in the development of the snack market focuses on the use of innovative technologies such as low-temperature or hybrid processes that allow the preservation of native ingredients of raw plant materials. In addition, the high antioxidant potential of, for example, chokeberry fruit can be used to support technological processes and create new products. The aim of the study was to evaluate the possibility of using chokeberry juice concentrate as a component of an osmotic solution to enrich apple samples with natural bio-ingredients and obtain dried apples with increased content of ingredients with antioxidant properties; pro-healthy apple chips. The research material consisted of apples that underwent osmotic dehydration in solutions of sucrose or sucrose and chokeberry juice concentrate and then were dried by the freeze-drying or the hybrid method. The freeze-drying was more beneficial for maintaining the vitamin C content, while the use of the hybrid method resulted in the preservation of more polyphenolic compounds. The sensory evaluation indicated the need to modify the composition of the osmoactive solution. Due to the use of chokeberry juice concentrate, the content of vitamin C, polyphenols, and the antioxidant activity of dried apples was increased.

## 1. Introduction

Increasing consumer awareness aimed at the impact of nutrition on health and proper functioning of the body, has prompted food producers to offer food that is not only safe, but also functional, containing, among others, pro-health ingredients such as polyphenols, vitamins, and minerals [1]. Technologies have been developed to meet these expectations by minimizing the use of “artificial” additives, striving for a “clean label”, and enriching products with ingredients such as polyphenols, vitamins, including those with antioxidant properties, e.g., vitamin C [2,3].

As demonstrated by Ferretti et al. [4], apples are the most consumed fruit, both fresh and processed. Apples are low in calories, and depending on the variety, they are a source of minerals, vitamins, and dietary fiber. The fruit is also a source of polyphenols, mainly procyanidins, which account for about 50% of the total amount of antioxidant compounds present in the fruit. Apart from procyanidins, apples also contain anthocyanins, phenolic acids, quercetin glycosides, and chalcones [5]. Due to new storage methods, fresh apples are available all year round. However, high competition and consumer expectations encourage producers to expand their offer with new, convenient products that are not only sensory acceptable but containing ingredients that have beneficial effects on human health.

Seasonal and microbiologically unstable products are subjected to various methods to extend their shelf life and availability to the consumer. One of such methods is drying. Various drying methods are used, ranging from drying in natural conditions (solar heating) through to convection, vacuum, and microwave or freeze-drying methods [6]. Each of these methods has its advantages and disadvantages. That is why many studies and research are aimed at developing a hybrid method that allows obtaining dried material with the expected physicochemical, microbiological, and sensory properties, taking into account energy savings [7]. Freeze drying is considered to be one of the best approaches to preserving the properties of dried products, especially labile or antioxidant compounds. However, it is still an expensive method that requires a freezing step, as well as a multi-hour and highly energy-consuming drying step [8]. An alternative to freeze-drying can be hybrid drying, combining convection, microwave, and vacuum drying. Additionally, osmotic dehydration before drying is one of the methods used to limit the negative changes that occur during drying by preserving bioactive ingredients or by enriching the product with additional ingredients (vitamins, antioxidants) [9]. Fruit juices, their concentrates, or pomace extracts are increasingly used instead of the commonly used sugar or salt solutions [10].

Chokeberry is a valuable fruit for the food and pharmaceutical industries due to its content of antioxidant compounds, primarily anthocyanins [11]. Chokeberry anthocyanins have strong antioxidant, antibacterial, anti-cancer, and anti-inflammatory properties [12,13]. Chokeberry is characterized by a high concentration of tannins, which on the one hand, have an anthocyanin stabilizing effect, but at the same time, are responsible for the intense vinegar taste of the fruit. It is because of the taste that chokeberry fruit is most often consumed in processed form (jams, juices). The content of vitamin C in chokeberry fruit is not high; it ranges from about 2.5 to about 13.0 mg in 100 g of fruit [11]. Its content depends, among others, on the date of harvest and the degree of maturity, as well as light intensity during the growth period. Due to its properties, chokeberry can be used as an additive to an osmotic solution, mainly to supplement ingredients that may be partially lost during technological processes or food enrichment, e.g., to obtain dried fruit with an increased content of bio-components [14].

Fruits and vegetables are the sources of vitamin C in the human diet [15]. Ascorbic acid is an important antioxidant. It increases resistance to some bacterial and viral diseases thanks to the participation in the production of immunoglobulins. It increases the absorption of calcium and iron, including non-haem iron from plants, so it is important in the treatment of anemia. It affects the ripening of collagen, vascular endothelium, increases the concentration of HDL, which inhibits the formation of atherosclerotic plaque. The addition of vitamin C is used in drinks and juices to compensate for the losses caused by the storage and processing of raw materials. Vitamin C is one of the least resistant of all vitamins, especially to high temperature and oxygen [16,17].

Polyphenols are secondary metabolites of plants, classified as non-nutritional components of food, however, in the human diet, they have a beneficial prophylactic effect on health [18,19,20]. Polyphenolic compounds have an antioxidant mechanism that is multidirectional and may rely on chelating metal ions, reducing properties, catalyzing the reaction of free radicals, or directly reacting with free radicals [21]. Polyphenols affect the tastiness and appearance (color, texture) of fruit and vegetables and the products made from them. Fruit containing anthocyanins have a characteristic red or blue color. Polyphenols, and especially anthocyanins, are very sensitive to high temperature and oxygen. Under their influence, they degrade, although they can also condense into complex compounds, e.g., tannins [22].

The aim of the study was to evaluate the possibility of using the chokeberry juice concentrate as a component of an osmotic solution to enrich apple samples with natural bio-ingredients and to obtain dried apples with an increased content of ingredients with antioxidant properties; pro-healthy apple chips. The scope of the work included the analysis of the content of dry matter content, vitamin C, total polyphenols content, the ability of extracts to scavenge DPPH radicals and the sensory evaluation of osmotically dehydrated and dried products, including control samples (without osmotic dehydration).

## 2. Results

An Idared apple variety was dehydrated in various solutions, as well as dried using the freeze-drying and hybrid method. The obtained droughts were analyzed, and the dry matter content, vitamin C content, total polyphenols, and antioxidant activity were determined. Dried apples were also subjected to a sensory evaluation. The aim of the research was to evaluate the effect of the type of osmotic solution and the drying method on the tested quality indicators of dried apples.

**Figure 1:**
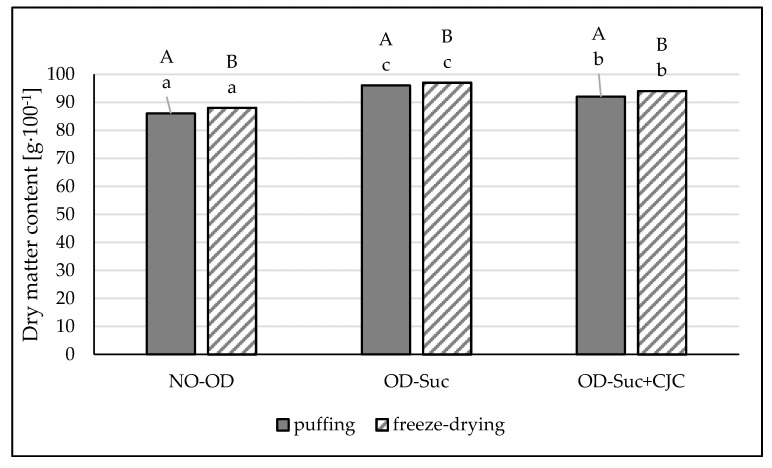
The influence of the type of osmotic solution and drying method on the dry matter content in apple samples dried by “puffing” or freeze-drying way; no pre-treated (NO-OD), pre-osmotic dehydrated in sucrose (OD-Suc) or in sucrose with chokeberry juice concentrate solution (OD-Suc + CJC). The same letter, a, b, c indicates a lack of statistically significant differences in the type of osmotic solution and A, B in the drying method.

The dry matter content in fresh apples was approximately 14.0 g·100 g^−1^. As expected, the samples dehydrated in sucrose solution were characterized by the highest values of dry matter (Figure 1). About 3–4 percentage points lower dry matter content was found in samples dehydrated in sucrose solution and chokeberry juice concentrate. A statistically significant influence of the osmotic dehydration and the type of osmotic solution on the dry matter content was demonstrated. However, no statistically significant differences between the data depending on the drying method used were found (Figure 1).

**Figure 2:**
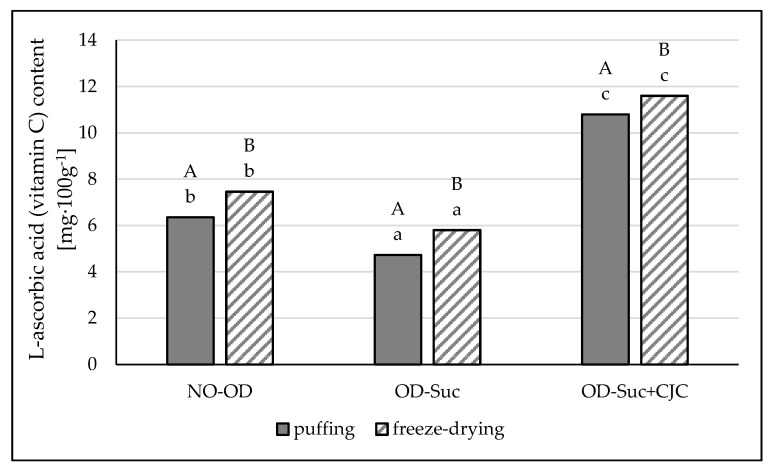
The influence of the type of osmotic solution and drying method on the vitamin C content in apple samples: dried by “puffing” or freeze-drying way; no pre-treated (NO-OD), pre-osmotic dehydrated in sucrose (OD-Suc) or in sucrose with chokeberry juice concentrate solution (OD-Suc + CJC). The same letter, a, b, c indicates a lack of statistically significant differences in the type of osmotic solution and A, B in the drying method.

The content of vitamin C in fresh apples was determined as 8.81 mg·100 g^−1^ (Figure 2). A significant influence of both the initial osmotic dehydration and the drying method on the content of this component was demonstrated. Dehydration in sucrose solution resulted in a reduction of the vitamin C content from about 34 to about 46%. Higher losses were found in the dehydrated and puffed dried samples. Compared to the application of the osmotic pre-treatment, the control samples, i.e., those dried but not previously subjected to osmotic dewatering, were characterized by a higher vitamin C content, by about 22 and 25%, respectively, after freeze-drying and puffing. The results of apples dehydrated in solution with the use of chokeberry juice concentrate were different. These samples were characterized by a significantly higher content of vitamin C, by about 23 and 32%, respectively, compared to the fresh apple and about two times higher than the dried samples previously dehydrated in sucrose solution. A similar tendency was shown in all dried apples, indicating a significantly higher degradation of vitamin C due to hybrid drying.

**Figure 3:**
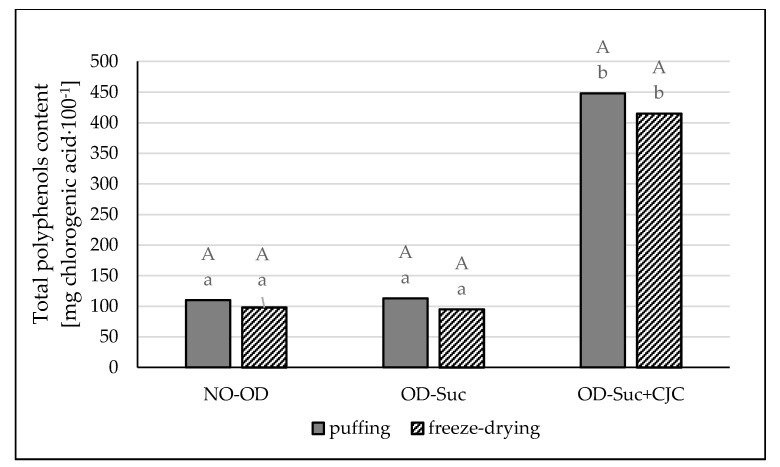
The influence of the type of osmotic solution and drying method on the total polyphenols content in apple samples: dried by “puffing” or freeze-drying way; no pre-treated (No-OD), pre-osmotic dehydrated in sucrose (OD-Suc) or in sucrose with chokeberry juice concentrate solution (OD-Suc + CJC). The same letter, a, b, c indicates a lack of statistically significant differences in the type of osmotic solution and A, B in the drying method.

The content of total polyphenols content in fresh apples was determined at the level of 83 mg of chlorogenic acid per 100 g of the product, while in the chokeberry juice concentrate 9870.60 mg of chlorogenic acid per 100 g of product. In samples dried without initial osmotic dehydration and dehydrated in sucrose solution and then dried, the content of polyphenols was determined at a similar level; statistical analysis showed no significant differences (Figure 3). The use of chokeberry juice concentrate as a component of the osmotic solution resulted in an over five-fold increase in the content of polyphenols. Moreover, it was shown that the convection–microwave–vacuum drying method allowed for a greater preservation of phenolic compounds in dried apples by approx. 8.0% (diagram 3) (Figure 3).

**Figure 4:**
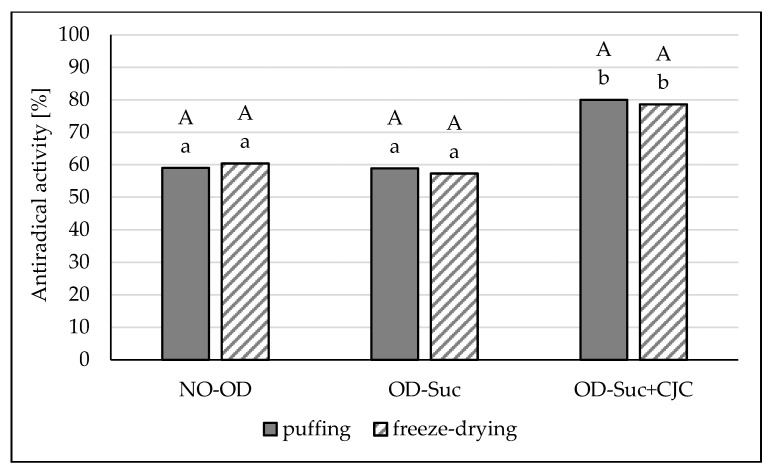
The influence of type of osmotic solution and drying method on the antiradical activity in apple samples: dried by “puffing” or freeze-drying way; no pre-treated (NO-OD), pre-osmotic dehydrated in sucrose (OD-Suc) or in sucrose with chokeberry juice concentrate solution (OD-Suc + CJC). The same letter, a, b, c, indicates a lack of statistically significant differences in type of osmotic solution and A, B in drying method.

Initial osmotic dehydration of the fruit in a sucrose solution and chokeberry juice concentrate had a significant effect on the anti-free radical activity compared to the use of a sucrose solution without CJC (Figure 4). However, it was shown that in the dried samples, the antiradical activity was significantly higher than in the raw material, but the drying method had no effect on the ability to scavenge DPPH radicals in the samples obtained by both drying methods. The antioxidant activity against DPPH radicals in fresh apples was approx. 57.0%, and in the dried apples 58.9–60.4%. On the other hand, dried apples dehydrated in a solution containing sucrose and chokeberry juice concentrate showed activity at the level of 78–80%. Samples dried with the puffing method (convection–microwave–vacuum) were characterized by slightly higher activity, by approx. 2% (Figure 4).

A general sensory assessment was performed on dried apple samples by the freeze-drying and hybrid (convection-microwave-vacuum) method with and without preliminary osmotic dehydration in a sucrose or sucrose solution and chokeberry concentrate mixture. The assessment was made on a linear scale ranging from 1 to 5 with specific boundary conditions: 1—unacceptable, 5—the most acceptable.

**Figure 5:**
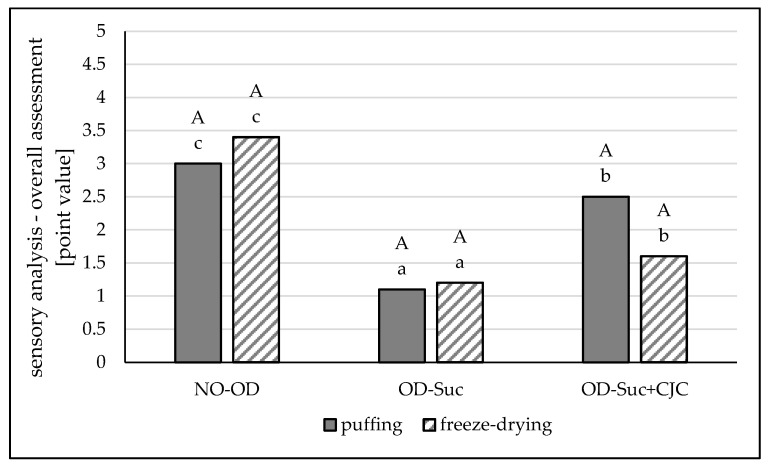
The influence of type of osmotic solution and drying method on the overall assessment for sensory evaluation in apple samples: dried by “puffing” or freeze-drying way; no pre-treated (NO-OD) and pre-osmotic dehydrated in sucrose (OD-Suc) or in sucrose with chokeberry juice concentrate solution (OD-Suc + CJC). The same letter, a, b, c indicates a lack of statistically significant differences in type of osmotic solution and A, B in drying method.

On the basis of the obtained evaluations, it was shown that the least desirable (1.1–1.2 points) were fruit samples dried after preliminary dehydration in sucrose solution. All evaluators indicated them as too sweet, even unacceptable. There were no statistically significant differences in these assessments in terms of the drying method used (Figure 5). The samples dehydrated in sucrose with chokeberry juice concentrate and then freeze-dried received an average score of 1.6 points. The taste of these samples was considered too bitter. In this study, apples dehydrated in a solution of sucrose and chokeberry juice concentrate and dried by the convection-microwave-vacuum method obtained a higher score, 2.5 points. This rating was about 1.5 points lower than in the case of apples dried by the freezing method without osmotic dehydration and by more than 0.5 points lower than in the case of dried apples that had not been previously dehydrated. The panelists found that these samples had a slightly sweet and slightly sour taste and a desirable, acceptable raw material-like flavor.

## 3. Discussion

The water content (dry matter content) of a food is one of the main factors affecting a product’s storage stability, but also its texture and consumer acceptability. It is possible to reduce the water content and thus extend the shelf life of the product by using various drying method. As Samborska et al. [23] demonstrated in their research, unfavourable changes in dried products, such as shrinkage, color change, and the loss of labile compounds, can be reduced by using preliminary osmotic dehydration.

Additionally, during osmotic dehydration, the product can be enriched with compounds lost in high-temperature processes, e.g., vitamins and phenolic compounds [10,24]. Lech et al. [25] proved in his research that osmotically dehydrated products are sensory attractive to consumers. Moreover, these products have a longer shelf life and retain their chemical and organoleptic properties better than fresh fruits, as demonstrated by Silva et al. [26]. In this study, the effect of osmotic dehydration and the type of applied osmotic solution on the water (dry matter) content of dried apples was demonstrated. Sharif et al. [24] reached similar conclusions in their research. They investigated the use of fruit juice concentrates as osmoactive agents on the properties of apples.

They showed that the composition of the concentrates to influence the osmotic pressure, diffusion and mass transfer during osmotic dehydration, including the amount of flow of the water removed and the solid gain of the dehydrated material. The current research showed statistically significant differences in dry matter content depending on the drying method used. However, the values of this index differed to a small extent, 86–88% in the control samples (without osmotic pre-treatment) and 92–97% in the osmotically dehydrated and dried samples. Therefore, it can be assumed that both the high-energy and cost-intensive freeze-drying method and the hybrid method used (puffing combining convection, microwave and vacuum drying) enable high dry matter contents to be obtained.

According to Kunachowicz et al. [27] and Płocharski et al. [28] the average vitamin C content in fresh apples is about 9.2 mg%. The content of this vitamin in fruits depend on many factors such as variety, pH, enzyme content and activity, cultivation method, harvest time, degree of maturity and sunlight, as well as time and storage conditions [29,30]. Vitamin C is relatively labile and therefore many studies on nutritional processes include it as an indicator of food quality [31]. In this study, losses of vitamin C in control samples, dried without osmotic dehydration, and higher, in samples using dehydration in sucrose solution, were shown. These losses may have been the result of elevated temperature, light, oxygen, and mass transfer in the dewatering process.

A greater loss of vitamin C was shown in the samples dried by the puffing method, although losses were also noted in lyophilisates. These results confirm the lability of vitamin C. The greater the number of operations, especially thermal ones, the greater the degradation of vitamin C [32,33]. The increase in vitamin C content in pre-dehydrated samples in sucrose solution contained chokeberry juice concentrate results from the diffusion of components between the two media (fruit sample, osmotic solution). In addition, the parameters used during osmotic dehydration (60 °C for 120 min) allow the preservation of labile ingredients, including vitamin C. However, despite statistically significant differences, the content of L-ascorbic acid in both droughts is quite similar; they differ by about 1 mg, therefore hybrid method is very useful and gives a higher dose of vitamin C compared to other traditional methods. This is probably due to the fact that the drying time and, at the same time, the action of the higher temperature was very short, lasting several minutes. In addition, the reason may be the increased content of vitamin C in the chokeberry juice concentrate and the effect of penetration the samples of other substances, mainly polyphenols, causing, among others, lowering the pH. Hence the protective effect of the concentrate. According to Kaliś [33], low temperature, blanching, freezing or alcoholic fermentation can slow down the degradation of vitamin C, and also preserve its antioxidant activity.

This explains the lower loss of vitamin C in the freeze-dried samples. The use of chokeberry juice concentrate as a component of the dehydrating solution increased the content of vitamin C. Even though chokeberry fruit is not a rich source of this vitamin (<13 mg%), the use of chokeberry juice concentrate allowed to increase its content in the tested dried apple samples. Średnicka-Tober et al. [34] obtained similar results. The possibility of using fruit juices as an osmotic solution was also described by Sharif et al. [24], who showed that the use of orange juice concentrate as an osmoactive solution increased vitamin C in candied apples.

Rząca and Witrowa-Rejchert [35] compared dried fruit obtained with different methods and showed that the best quality and nutritional parameters were found in freeze-dried samples, but the dried fruit obtained by puffing was rated higher by consumers than that obtained by convection drying. The studies by Gamboa et al. [36] showed that the greatest losses of vitamin C occur in the first hour of drying and it is related to the high water content. They pointed out that the degradation of vitamin C content was less dependent on the action of high temperature, while showing high vitamin C retention at lower temperatures of 40–50 °C, regardless of the duration of the process. Other factors mentioned above (access of oxygen, light) also have a significant impact on the degradation of vitamin C [33]. According to the provisions of Regulation 1169/2011 [37], the recommended daily reference dose of vitamin C by man is 80 mg. Quantities determined in dried apples dehydrated in a solution with the addition of chokeberry juice concentrate amounted to about 10.8–11.6 mg%.

Consumption of 100 g of such snacks is able to cover less than 14% of the recommended dose. This means that the consumer should consume other products containing vitamin C to cover the recommended reference intake doses. This information also provides the basis for further research aimed at increasing or preserving more vitamin C in the developed apple bio snacks.

Another nutritional important component of the resulting apple snacks are polyphenols. Many studies show that freeze-drying influences the preservation of the original composition of the raw material to a greater extent [35,38], which has not been confirmed in the current research. Polyphenols are compounds which, under the influence of external factors, e.g., high temperatures can degrade, but can also form new phenolic compounds or transform into complex tannins. Therefore, the action of higher temperature during the puffing process could initiate the formation of polyphenolic compounds as well as their condensation, as evidenced by the higher content of the determined compounds in samples subjected to hybrid drying. Moreover, as shown by Kaliś [33] in his research, increased temperature may increase the antioxidant effect of vitamin C, which in turn may increase the overall content of antioxidant compounds in the food matrix. Initial freezing and then removal of the frozen water by sublimation under reduced pressure at a temperature of 25 °C protect polyphenolic compounds.

Simultaneously, large temperature differences, as well as long processing times (freeze-drying—24 h), may result in losses of these compounds. The degradation of polyphenolic compounds is specific for each product and depends on many factors, such as storage temperature, oxygen availability, water activity, pH, enzymes, applied technological treatment [39]. As the research conducted by Udomkun et al. [40], an increase in the drying temperature may result in higher antiradical activity and a higher content of total polyphenols. Safafar et al. [41] implied that the antioxidant activity in dried papaya samples is not affected by phenolic compounds alone. The authors have said other constituents, carbohydrates, tocopherols, carotenoids, terpenes, and pigments, probably contribute to the total antioxidant activity as well. The results obtained in this study confirm that microwave-vacuum drying can be successfully used to obtain dried fruit with a nutritional value comparable to the use of freeze-drying. That is why it is so important to determine the parameters of technological processes, as well as the storage conditions of finished products, in order to ensure microbiological safety, focus on the preservation of ingredients that have a beneficial effect on the consumer’s body, and even protect against diseases.

According to De Bruijn and Bórquez [42], the hybrid method makes it possible to obtain a sensory-attractive product with extended durability, while maintaining features such as color and microstructure. In addition, microwave-vacuum drying reduces heat and oxygen exposure, leading to higher retention of vitamins and phenolic compounds, which are generally sensitive to thermal effects and oxidation, as shown by Chandrasekaran et al. [43]. The preservation of as many antioxidant compounds as polyphenols as possible is one of the goals of modern technology. As numerous studies have shown, regular and long-term consumption of products rich in polyphenols protects the human body against certain cancers, cardiovascular diseases, type 2 diabetes, osteoporosis, pancreatitis, gastrointestinal problems, or lung damage [44]. 

The content of antioxidant compounds, including polyphenols, is closely related to antioxidant activity. It is this indicator that shows how phenolic compounds can have a positive effect on the human body. In this study, the activity of polyphenols against stable DPPH radicals was determined. In accordance with the methodology used, the absorbance of the sample was examined 30 min after the addition of individual reagents, and then the relationship between the control sample (without extract) and the proper sample was expressed as a percentage, taking into account the influence of the reagents through the blank test. As confirmed in their research by Choo et al. [6], the content of polyphenols, as well as the antioxidant activity, depends on the temperature of the drying process or the microwave power. The increase in temperature from 40 to 60 °C resulted in a decrease in antioxidant activity by about 18–29%, depending on the method of determination used. The same authors showed the oxidative degradation of polyphenols along with the prolongation of exposure to high temperature on the sample. Nowacka et al. [45] showed the smallest loss of antiradical activity in freeze-dried samples in comparison to other drying methods. Choo et al. [6] came to slightly different conclusions. The authors showed the highest antioxidant activity of freeze-dried samples, which resulted from the applied low process parameters and protection of temperature-sensitive compounds. At the same time, the team showed the smallest loss of antioxidant capacity in the microwave-dried samples.

One of the important factors guaranteeing the interest and demand for the offered food is its sensory character. Customers pay more and more attention to the health-promoting properties of the food they buy, but the aesthetics on the plate is still an important factor of consumer preferences. Chokeberry is a fruit with a distinct astringent flavor that is difficult to accept when consumed. Therefore, despite its beneficial properties, this fruit is not consumed fresh, but in processed form. Scientists have used this fruit to enrich others, especially dried products. Kowalska et al. [46] investigated the possibility of using chokeberry juice as a component of a dehydrating solution. As in this study, products dehydrated in chokeberry juice did not meet with consumer acceptance, and the analyzed products received low marks. The influence of drying method on the sensory feature of apples, which is color, was analyzed by Michalska et al. [47]. They showed that freeze-dried apples were characterized by the lightest in color (L*), while samples dried using the microwave-vacuum method had the highest value of yellow pigment (b* index). The consumer expects a processed product with characteristics similar to or better than the raw material. De Castro et al. [48] confirmed the influence of osmotic dehydration on the properties of guava, at the same time showing the influence of the temperature used on the color of the obtained droughts and thus on the sensory properties and consumer acceptability. In the conclusions, the authors stated that the dehydrated fruit retained its color while drying, especially of products containing a higher sugar content, resulting, among others, from dehydration in a sucrose solution, were characterized by a much darker color, which in relation to some fruit is not acceptable for the consumer.

The shaping of sensory features is the result of both the raw material composition and the processes that the product undergoes. Sensory characteristics are also related to the content and composition of the polyphenolic compounds present in the product. For example, the presence of anthocyanins affects the purple color of fruit and vegetables, and tannins are responsible for the tart taste. During technological processes, especially high-temperature processes, changes occur in the content and composition of polyphenols, which generates, among others, precursors of taste, smell, or color. The main and dominant polyphenols present in chokeberry fruit are anthocyanins, especially cyanidin-3-*O*-glucoside. Moreover, chokeberry fruit is a source of flavonols, proanthocyanidins, and phenolic acids and dietary fiber, vitamin B, carotenoids, tocopherols, vitamins C and K, as well as the elements K, Ca, P, Mg and Na, Mn, Si, Ni, and B. All these ingredients affect the quality of chokeberry fruit. However, the anthocyanin content has the greatest influence on the taste and color of chokeberry. As a result of high temperature, phenolic compounds, especially anthocyanins, which are very sensitive to temperature, are degraded. However, the mechanism of shaping sensory features is more complex. As demonstrated by Sidor et al. [49] the addition of ascorbic acid reduces the cyanidin content in the product. The same authors also showed interactions between the anthocyanins present in chokeberry and added sugars, e.g., sucrose, which resulted in a reduction in the content of the determined phenols. Higher temperature also causes evaporation of volatile compounds, e.g., acids, which are responsible for the taste of the products. The sensory analysis of apple chips tested in this study, expressed as a general evaluation, showed lower acceptability of freeze-dried samples. This may have been due to the lower losses of anthocyanins responsible for the dark color and sour taste of the product. In the hybrid method, the process was carried out at a higher temperature, which could affect the decomposition of anthocyanins and evaporation of some volatile acids, for the sour, tart taste of the fruit. In addition, microwave treatment (one of the hybrid drying steps) can facilitate the release of other bioactive compounds from the apple cells/cell walls.

Corollaro et al. [50] confirmed the significant importance of sensory characteristics, especially those related to texture aspects, on consumer preferences. The results of the sensory assessment in this study indicate the direction of further research aimed at meeting customer expectations.

## 4. Material and Methods

### 4.1. Material

The research material was firm and ripe similar-sized (7.5 ± 1.5 cm) diameter apples var. Idared, purchased directly from the producer (fruit farm, Grójec, Poland). The fruit was harvested in September 2018, stored in refrigerated conditions (controlled atmosphere) at the grower. Apples were used for the research in the period January–April 2019; they were bought from the producer, on average every 2 weeks. Next, the fruit was stored in refrigerated conditions until analysis at temperature of 5–6 °C and about 85% air humidity. The apples were cut into slices about 10 mm thick in a Robot Coupe chopper (CL50 STALGAST 713500, Stalgast Ltd., Radom, Poland) and then manually with a sharp knife into cubes of 10 mm. Afterwards, they were immersed in 1% (1 g·100 g^−1^ H_2_O) citric acid for 10 min at 25 °C.

### 4.2. Osmotic Dehydration

The osmotic solution was used in the form of a 50°Brix aqueous solution made of sucrose and chokeberry juice concentrate. The sucrose was purchased at a local store and the chokeberry juice concentrate (var. *Aronia melanocarpa*, 65°Brix) was purchased from RAUCH Poland. An aqueous solution of sucrose with a concentration of 50% was prepared and the chokeberry juice concentrate was diluted to 50% and then both were mixed in a 1:1 ratio. The process temperature was kept constant at 60 °C for 120 min [51].

### 4.3. Drying

Freeze drying was preceded by freezing the fruit at −40 °C for about 4 h. The samples were then dried in a drying chamber at 63 Pa, at 25 °C hot plate temperature for 24 h (24 h). Drying with the “puffing” effect was carried out in two stages. In the first stage, the samples were dried by convection for 3 h at a drying air temperature of 50 °C and an air velocity of 2 m/s. The second stage was microwave-vacuum drying. The process temperature was 70 °C, and the drying time was 6 min. The microwave power was set at 400 W, and the pressure 3.5 kPa. The dried samples were packed in plastic bags, which were sealed. Until the analyzes were carried out, the apple samples were stored in a dry place, out of the light.

### 4.4. Chemical Analyses

All analyses were performed at least in three parallel replicates for fresh, osmo-dehydrated, dried (with or without osmotic pre-treatment) by freeze-drying and convection-microwave-vacuum drying methods.

### 4.5. Dry Matter Content 

Dry matter content of each tested sample (fresh, osmo-dehydrated, and dried) was determined gravimetrically by vacuum drying (HORYZONT SPT 200, Krakow, Poland) at ≤100 mmHg and a temperature of 70 °C until a constant weight has been achieved. The samples were weighed on an analytical weight with the accuracy of 0.001 g [52].

### 4.6. Determination of Vitamin C (L-Ascorbic Acid) Content

The content of vitamin C in apples was made by spectrophotometric method based on the absorbance at 500 nm [53]. The spectrophotometer was zeroed using xylene. A calibration curve was also prepared from 5 mL of oxalic acid, 5 mL of buffer solution and in succession: 0.2; 0.4; 0.6; 0.8; and 1.0 mL solution of 2,6-dichloroindophenol and xylene. The standard curve was determined in two parallel replicates. Based on the results obtained, a curve of absorbance versus volume of a given dye solution was drawn. The excess volume of 2,6-dichloroindophenol was read from the curve, which was added to the test sample, which corresponded to the absorbance value of this sample. Results were expressed in mg acid/100 g.

### 4.7. Determination of Total Polyphenols Content by the Folin–Ciocalteu Method

Total polyphenol content was determined by Folin–Ciocalteu’s method [54]. Based on preliminary tests, a 70% acetone solution was used as a solvent to prepare extracts. The extracts were prepared by weighing about 2 g of fresh apples and about 0.2 g dried apples into 300 mL grinding conical flasks and adding 100 mL of 70% acetone (*v*/*v*). The samples were then shaken for 30 min (at ambient temperature) in a Multi-Shaker PSU 20 Biosan shaker. Following this procedure, the solutions were filtered through the corrugated filters into 100 mL grinding flasks. In order to determine the total polyphenol content, 300 µL of the extract was taken from the tubes, and 4.15 mL of deionized water, 500 µL of 20% sodium carbonate solution, and 50 µL of the Folin–Ciocalteu reagent were added. The blank sample was prepared by sampling: 300 µL of the extraction solution, 4.15 mL of deionized water, 500 µL of the 20% sodium carbonate solution, and 50 µL of the Folin–Ciocalteu reagent. Absorbance was measured at 700 nm on a Shimadzu UV-160A spectrophotometer. The apparatus was zeroed to a blank. In order to calculate the total polyphenol content, a standard curve was prepared. The standard curve was plotted for chlorogenic acid for various concentrations (0, 25, 50, 75, and 100 µL) used in absorbance measurements. Based on the results obtained, the graphical dependence of the absorbance of the solution on the amount of chlorogenic acid contained in it was plotted. The total polyphenol content was calculated on the basis of the calibration curve and expressed in chlorogenic acid equivalent in mg per 100 g d.m. (mg·100 g^−1^) of the product. Two extracts from each mass were made, and polyphenols content was determined in three parallel repetitions for each extract. The average of six repetitions for each mass was considered the final result.

### 4.8. Determination the Ability of Extracts to Inactivate Stable DPPH Radicals

The antioxidant ability and DPPH• scavenging capability of apple extracts were determined using the modified method proposed by Wu [55]. The extracts were prepared analogously to determinations of polyphenol content. The basic DPPH• extract was prepared by dissolving 1.2 mg of DPPH• in 50 cm^3^ of 99% methanol, from which the blank sample was prepared. The absorbance measurement at a wavelength of 515 nm was carried using a The Heλios γ ThermoSpectronic spectrometer. The antioxidant activity of the analyzed extracts (A) was recalculated based on absorbance results of the exact and control samples in Heλios γ ThermoSpectronic spectrometer at a wavelength of 515 nm, against 99% methanol. The antioxidant activity of the apple extracts against DPPH• radicals was calculated based on results achieved for the control sample (absorbance of DPPH• solution) and exact samples (the ability of the substance tested to inhibit oxidation reaction) and expressed in %.

### 4.9. Sensory Evaluation

The sensory evaluation was carried out by a trained team of 20 panelists. The panelists made an overall assessment of the apple samples and marked the results on a scale ranging from 1 to 5, with specific boundary conditions. 1 meant the product was “unacceptable” and 5 was “acceptable and satisfactory”. The overall assessment was characterized as a general sensory impression combining all the taste, smell, and textural features. The panelists were asked to take into account quality features such as color, taste, aroma, and consistency in the overall assessment.

### 4.10. Statistical Analyses

The statistical analysis of results was performed using Statistica 12.0 software based on one- and two-way analysis of variance at a significance level of *p* = 0.05. Significant differences between means were determined using the Tukey test. The impact of the osmotic dehydration process, the type of osmoactive solution, and the drying methods on the antioxidant and sensory properties of dried apples were determined.

## 5. Conclusions

Technological development, increasing consumer awareness of the impact of nutrition on health and attaching more and more importance to reducing food waste all affect the developmental direction of the food industry. Until recently, consumers expected sensory-interesting products with a long shelf life, which resulted in the trend of using various food additives, both artificial and natural. Today, the so-called clean label, minimizing the use of additional substances, while expecting a product that is convenient, quick to cook, or for direct consumption, showing broadly understood functional properties. To meet these expectations, snacks in the form of dried apples, enriched with natural ingredients, through the use of health-promoting properties of chokeberry, were offered. The use of osmotic dehydration in a solution containing chokeberry juice concentrate increased the content of vitamin C, the total amount of polyphenols, and the antioxidant activity of the snacks developed. However, only samples without initial osmotic dehydration and dried by freeze-drying and hybrid method were satisfactorily (3.0–3.4). Among the pre-dehydrated samples, the highest, although unsatisfactory (2.5), marks were awarded to samples dehydrated in a solution of sucrose and chokeberry concentrate mixture, dried by the hybrid method. Promising prospects for the use of the hybrid drying method (convection-microwave-vacuum) as a method allowing for the preservation of a large number of bio-compounds have also been shown. The dried apple snacks did not meet with much appreciation by the panelists for their sensory properties, possibly due to too sour, tart taste, and too dark red color. This is an aspect that requires elaboration, and the obtained results confirm the need to continue research.
